# Complex Aortic Valve Repair After En-Bloc Rotation of the Outflow Tracts: A Case Report

**DOI:** 10.1093/icvts/ivaf240

**Published:** 2025-10-10

**Authors:** Andrea Rapagnani, Gebrine El Khoury, Mona Momeni, Alain Jean Poncelet

**Affiliations:** Department of Cardiothoracic and Vascular Surgery, Université Catholique de Louvain, Cliniques Universitaires Saint-Luc, Brussels, 1200, Belgium; Department of Cardiothoracic and Vascular Surgery, Université Catholique de Louvain, Cliniques Universitaires Saint-Luc, Brussels, 1200, Belgium; Department of Anaesthesiology, Université Catholique de Louvain, Cliniques Universitaires Saint-Luc, Brussels, 1200, Belgium; Department of Cardiothoracic and Vascular Surgery, Université Catholique de Louvain, Cliniques Universitaires Saint-Luc, Brussels, 1200, Belgium

## Abstract

The En-bloc Rotation of the Outflow Tracts, as suggested by Anderson in 2016^1^, is a surgical approach for complex congenital heart defects, particularly in patients with transposition of the great arteries and associated ventricular septal defect and left ventricular outflow tract (LVOT) obstruction (LVOTO). Despite its effectiveness, concerns have arisen following a study by Stoica et al. (2022), which reported that 16% of patients developed at least moderate aortic regurgitation (AR) post-surgery. The mechanisms behind this complication remain unclear. This case report presents a 4-year-old patient who developed significant AR (grade 3+/4), with left ventricular dilation (LVEDD: 43 mm), 3 years after an En-bloc Rotation, necessitating aortic valve repair. Initial echocardiography showed trivial AR, with progression of AR within 18 months and associated annular dilation (24 mm). The repair procedure involved plication of the LVOT, annuloplasty, and leaflet adjustments. At discharge, residual AR was mild to moderate (1+/4). Seven months later, echocardiography revealed stable left ventricular dimensions and moderate AR (grade: 2+/4). This case emphasizes the need for early detection and intervention for AR in patients after En-bloc Rotation surgery. Further research is needed to identify predisposing factors and refine surgical strategies.

## INTRODUCTION

The En-bloc Rotation of the outflow tracts is an established technique for repairing TGA with VSD and LVOTO, as well as double outlet right ventricle with subpulmonary VSD and LVOTO. A multicentre study by Stoica et al^2^ showed that 16% of patients (4/25) developed moderate or worse AR, while only 4% (1/25) required LVOT reintervention.

The factors for the development of moderate to severe AR after En-bloc Rotation remain unclear. This contrasts with the arterial switch procedure, which has been associated with predisposing factors for neoaortic regurgitation (prior pulmonary artery banding, VSD, Taussig-Bing, mild preoperative pulmonary regurgitation, concomitant relief of LVOTO, and more than mild neo-AR at hospital discharge).^3^^,^^4^

This case report presents one of the 27 patients described in the study by Stoica et al^2^ operated in 2020, who developed grade 3+/4 AR and subsequently underwent aortic valve repair (AVR). Parental consent was obtained for publications and submitted pictures.

## CASE PRESENTATION

A 4-year-old patient, weighing 16 kg, had undergone an arterial En-bloc Rotation of the outflow tracts at 11 months in 2020. The repair included LVOT enlargement, pulmonary valve repair (commissurotomy), and closure of both an atrial septal defect and a VSD with a Lecompte manoeuvre. Despite the initial absence of AR, the patient developed mild AR (grade 1+/4) within the first year, progressively worsened, concomitant with annular dilation (23 mm by year 2, Z Score +9).

Four years after the initial procedure, transthoracic echocardiography (TTE) showed grade AR 3+/4 associated with LV dilatation (LVEDD 43 mm,+3.8ZSc and LVESD 30 mm,+4.2ZSc). The decision was made to proceed with surgical intervention due to the risk of left ventricular dysfunction.

## AORTIC VALVE REPAIR

Intraoperative TEE confirmed severe AR (3+/4), with a central jet partially excentric (left and posterior leaflet prolapse) and annular dilatation (25 mm, *Z*-score + 9.8). Left ventricle ejection fraction (EF) was preserved (55%). Aortic root size was normal (16 mm, *Z*-score + 1.3). (Video 1)

After resternotomy and cardiopulmonary bypass via aortic and bicaval cannulation, the main pulmonary artery was transected to access the aortic root. Intraoperative inspection revealed: trileaflet valve with normal anatomical arrangement of the cusps and coronary artery origins, thickening of all 3 free margins, mild prolapse of the posterior leaflet, severe retraction and prolapse of the left coronary leaflet secondary to asymmetric dilation of the LVOT, markedly pronounced at the level of the closure zone of the previous VSD.

## REPAIR TECHNIQUES

**Figure 1.**
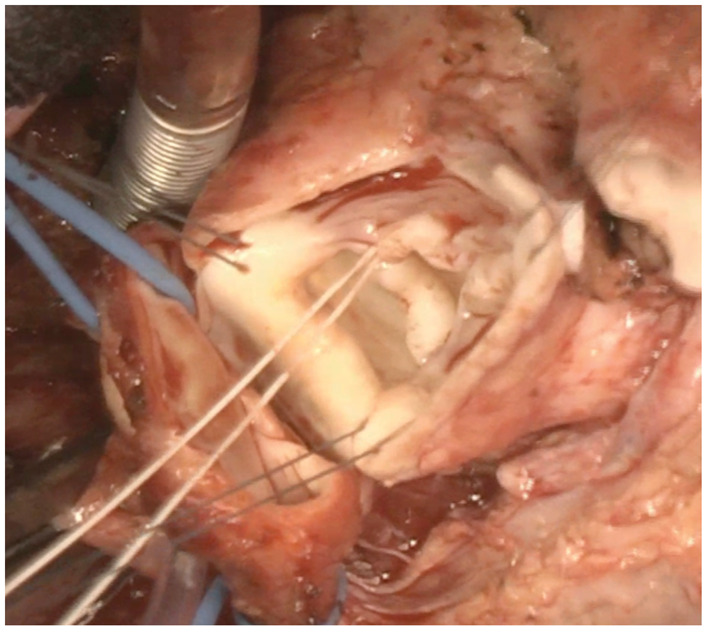
AVR Technique

Horizontal LVOT plication at the VSD patch level was performed using Gore-Tex/4–0. An internal/external annuloplasty was carried out at the virtual basal ring, with Gore-Tex/4–0 suture, calibrated to 15 mm (Hegard dilator). Leaflet free margins were shaved, and 2 sub-commissural plication sutures were placed at the posterior-left and left-right commissures to enhance coaptation (Figure 1). Additionally, the free margin of the left cusp was plicated to increase its coaptation height. (Video 1).

Intraoperative TEE showed good annuloplasty (15 mm), with mild residual AR (grade: 1+/4) and good biventricular function (Video 1).

## POSTOPERATIVE EVOLUTION

The patient remained haemodynamically stable and was extubated on postoperative day 1. He was discharged from the intensive care unit on postoperative day 3. A transthoracic echocardiogram on postoperative day 9 demonstrated normal valve function with mild residual AR (1+/4), a peak gradient of 10 mmHg, and a decreased left ventricular dilatation (LVEDD 39 mm, *Z*-Score + 2.2) with moderate EF reduction (43%). The patient was discharged home on postoperative day 11.

## FOLLOW-UP

Seven months postoperatively, the patient remained asymptomatic. Trans-thoracic echocardiography showed moderate AR (grade: 2+/4), annular size 17 mm (+2 mm), and borderline LV dimensions (LVEDD 42 mm, Z + 3.1; LVESVi 42 ml/m^2^; LVEF 52%).

## CASE DISCUSSION

En-bloc Rotation of the Outflow Tracts remains an effective approach for complex congenital heart conditions. This case highlights AR as a potential long-term complication. Despite the surgical challenges, the early surgical repair demonstrated significant early improvements in valve function, allowing left ventricular function recovery and delaying future aortic valve surgery.

The most recent follow-up underscores the role of annular dilatation in valve failure. Similar to adults with bicuspid and tricuspid aortic valves, a large basal ring can predict AR recurrence following AVR. With valve-sparing reimplantation, however, large basal rings do not predict recurrence, likely due to increased stability of circumferential annuloplasty. In adult patients, our group has previously recommended circumferential annuloplasty for basal rings ≥28 mm to reduce the risk of recurrence.^5^

True aorto-ventricular stabilization, as achieved with root reimplantation surgery, remains challenging and has not yet been reported in growing children.

In our patient, annuloplasty was calibrated on a Hegard dilator 15 mm, a diameter we thought would be large enough to allow appropriate patient growth. As the longevity of AVR mostly depends on the intrinsic mechanism(s), we anticipated that this patient would ultimately require a formal aortic valve replacement.

Future studies should identify predisposing factors for AR and optimize surgical strategies. Monitoring long-term outcomes is crucial for refining approaches to complications.

## Data Availability

The data used in this case report were obtained from the hospital’s internal platform and are not publicly available due to patient privacy and data protection regulations. Interested researchers may request access to the anonymized data through the hospital’s archival system, subject to ethical approval and review by the relevant institutional review board.
